# Pumpkin CmHKT1;1 Controls Shoot Na^+^ Accumulation via Limiting Na^+^ Transport from Rootstock to Scion in Grafted Cucumber

**DOI:** 10.3390/ijms19092648

**Published:** 2018-09-06

**Authors:** Jingyu Sun, Haishun Cao, Jintao Cheng, Xiaomeng He, Hamza Sohail, Mengliang Niu, Yuan Huang, Zhilong Bie

**Affiliations:** Key Laboratory of Horticultural Plant Biology, Ministry of Education, College of Horticulture and Forestry Sciences, Huazhong Agricultural University, Wuhan 430070, China; Jingyu.sjy@gmail.com (J.S.); chsmugua@webmail.hzau.edu.cn (H.C.); chengjintao@mail.hzau.edu.cn (J.C.); xmh_hzau@163.com (X.H.); hamzadarbzu@gmail.com (H.S.); nfuyuer@163.com (M.N.); huangyuan@mail.hzau.edu.cn (Y.H.)

**Keywords:** grafted cucumber, pumpkin, salt tolerance, high-affinity potassium transporter (HKT), sodium transport

## Abstract

Soil salinity adversely affects the growth and yield of crops, including cucumber, one of the most important vegetables in the world. Grafting with salt-tolerant pumpkin as the rootstock effectively improves the growth of cucumber under different salt conditions by limiting Na^+^ transport from the pumpkin rootstock to the cucumber scion. High-affinity potassium transporters (HKTs) are crucial for the long distance transport of Na^+^ in plants, but the function of pumpkin HKTs in this process of grafted cucumber plants remains unclear. In this work, we have characterized *CmHKT1;1* as a member of the *HKT* gene family in *Cucurbita moschata* and observed an obvious upregulation of *CmHKT1;1* in roots under NaCl stress conditions. Heterologous expression analyses in yeast mutants indicated that CmHKT1;1 is a Na^+^-selective transporter. The transient expression in tobacco epidermal cells and *in situ* hybridization showed CmHKT1;1 localization at plasma membrane, and preferential expression in root stele. Moreover, ectopic expression of *CmHKT1;1* in cucumber decreased the Na^+^ accumulation in the plants shoots. Finally, the *CmHKT1;1* transgenic line as the rootstock decreased the Na^+^ content in the wild type shoots. These findings suggest that CmHKT1;1 plays a key role in the salt tolerance of grafted cucumber by limiting Na^+^ transport from the rootstock to the scion and can further be useful for engineering salt tolerance in cucurbit crops.

## 1. Introduction

Salinity affects approximately 7% of the world’s land including agricultural lands and is a crucial factor limiting over 30% of irrigated and 7% of dryland agriculture worldwide [1]. Na^+^ is a common salt ion that is toxic to most crops [2]. Regulation of Na^+^ influx, efflux, allocation, and compartmentation is important for plants to cope with Na^+^ accumulation in the shoots. This process involves a complex network of channels and transporters that participate in ion uptake and compartmentation in plant cells and tissues.

High-affinity K^+^ transporter-1 (HKT1) encodes a Na^+^ preferential transporter that principally controls root-to-shoot Na^+^ delivery via the withdrawal of Na^+^ from the xylem sap [3,4,5,6]. Members of the *HKT* family can be divided into two subfamilies, namely, *HKT1* and *HKT2*; the former subfamily functions as a Na^+^ uniporter, whereas the latter serves as a Na^+^ and K^+^ symporter [7]. HKT1 transporters are involved in retrieving Na^+^ from the transpiration stream [8,9,10] by mediating the influx of Na^+^ into the xylem parenchyma cells in the roots [3]. *HKT1* genes play a crucial role in Na^+^ transport in several plant species, such as *Arabidopsis*, rice, wheat, and maize [5,8,9,11,12,13,14]. AtHKT1;1 is localized at the plasma membrane of the xylem parenchyma cells in the shoots [10] and at the phloem tissues of both roots and shoots [11]. Therefore, AtHKT1;1 might be involved in Na^+^ recirculation from the shoots to roots via phloem and/or Na^+^ unloading from the xylem into xylem parenchyma cells [10,11]. An enhancer trap system overexpressing *AtHKT1;1* in the mature root stele of *Arabidopsis* decreases Na^+^ accumulation by 37–64% in the shoots [9]. These changes in the shoots are caused by the augmented influx of Na^+^ into stellar root cells mediated by AtHKT1;1, which reduces the root-to-shoot transfer of Na^+^ [9]. In rice, *AtHKT1;1* expression in the roots is affected by low Na^+^ concentration in the shoots [15]. In soybean, *GmHKT1;4* overexpression significantly enhances the tolerance of transgenic tobacco plants to NaCl stress by promoting K^+^ accumulation and inhibiting Na^+^ accumulation in the shoots [16].

Cucumber (*Cucumis sativus* L.) is an important vegetable crop worldwide, which is sensitive to salt stress [17]. Saline soils or saline irrigations often limit cucumber production [18,19,20,21,22]. Pumpkin is often used as the rootstock of cucumber, and it is relatively salt-tolerant [23,24,25]. Grafting onto salt-tolerant pumpkin rootstock can effectively improve the growth of cucumber plants grown under salt conditions [19,23,24]. Our previous study showed limited radial transport of Na^+^ to the stele by using pumpkin as rootstock, thereby restricting Na^+^ transport from the roots to shoots [26]. However, the function of pumpkin HKT in this process remains unclear.

In the present study, we characterized the pumpkin HKT gene *CmHKT1;1* and investigated its contribution to salt tolerance. The expression of *CmHKT1;1* in different tissues of pumpkin in response to NaCl treatment was examined. CmHKT1;1 localization was investigated by the transient expression of CmHKT1;1 in tobacco epidermal cells and by *in situ* hybridization to localize the transcripts of *CmHKT1;1* in pumpkin. The features of ion transport mediated by CmHKT1;1 were examined by heterologous expression systems in yeast mutants. To test the gene function in planta, we ectopically expressed *CmHKT1;1* in cucumber. We also constructed grafting combination by using the cucumber *CmHKT1;1* transformation line as the rootstock and tested its effect on leaf ion accumulation. Results demonstrate that *CmHKT1;1* is involved in the mechanism by which the pumpkin rootstock limits Na^+^ accumulation in grafted cucumber shoots.

## 2. Results

### 2.1. Identification and Characterization of Pumpkin CmHKT Genes

Based on the amino acid sequence of *Arabidopsis* HKT1 (AT4G10310.1), BLASTP research was used to identify orthologs of HKT proteins in the pumpkin protein database (http://cucurbitgenomics.org/organism/9). Two highly homologous sequences were discovered (CmoCh10G003830.1 and CmoCh10G003840.1), namely, CmHKT1;1 and CmHKT1;2.

The full-length amino acid sequences of CmHKT1;1 and CmHKT1;2 exhibited high similarity to those of AtHKT1 (68.67% and 64.76%). The open reading frame of CmHKT1;1 is 1461 bp in length and encodes a polypeptide of 487 amino acids. The open reading frame of CmHKT1;2 is 1245 bp in length and encodes a polypeptide of 415 amino acids.

**Figure 1:**
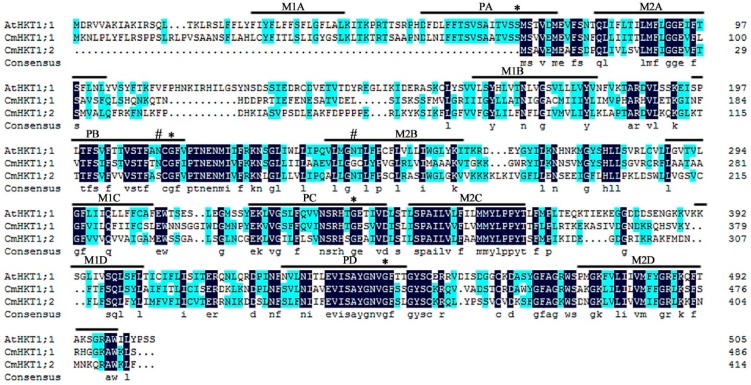
Multiple alignment of CmHKT1;1 and CmHKT1;2 amino acid sequences with *Arabidopsis thaliana* AtHKT1;1. Identical and similar amino acids are represented by dark blue and light blue, respectively. Sequences were aligned using DNAMAN (Lynnon Biosoft). Positions of the transmembrane and pore segments were predicted according to the model proposed for the topology of the AtHKT1;1 protein, which was based on the four-MPM structural model. The conserved Gly residues in the K^+^ channel selectivity filter GYG of the P-loop-like domains (highlighted by asterisks) determine the K^+^ selectivity of HKTs [5]. The presence of Gly in the PA-loop is conserved in K^+^-permeable HKTs (class II), whereas the presence of Ser instead of Gly is conserved in Na^+^-permeable HKTs (class I) [5,7]. “#” defines the positions of Asp residues (D) that are shown to be essential determinants for K^+^ transport activity in TsHKT1;2 [29].

In *Arabidopsis*, the protein structure of AtHKT1;1 is proposed to contain a four membrane-pore-membrane (MPM) (transmembrane segment, pore, and transmembrane segment) structural model [27,28]. To analyze the protein structure of the two pumpkin HKT proteins, we aligned the deduced amino acid sequences with the known AtHKT1;1 amino acid sequence. The positions of the transmembrane and pore segments were predicted according to the model for AtHKT1;1 (Figure 1). Similar to AtHKT1;1, CmHKT1;1 consisted of four MPM motifs, whereas CmHKT1;2 lacked the first MPM motif (Figure 1). The glycine residue in the first P-loop of HKT proteins is crucial for K^+^ selectivity and transport [5]; thus, the loss of integrity of the first P-loop affects CmHKT1;2 function. We further examined the *CmHKT1;2* transcript expression level in pumpkin roots under salt stress, and results showed that salt stress did not induce *CmHKT1;2* expression (Supplementary Figure S1A). Thus, we mainly focused on CmHKT1;1 in the analysis of protein function. The serine or glycine residues at the Na^+^-K^+^ selectivity filter positions in the PA, PB, PC, and PD regions in the CmHKT1;1 protein were the same as those in AtHKT1;1 (Figure 1), especially the serine at the PA region, indicating that CmHTK1;1 is a class I HKT. 

**Figure 2:**
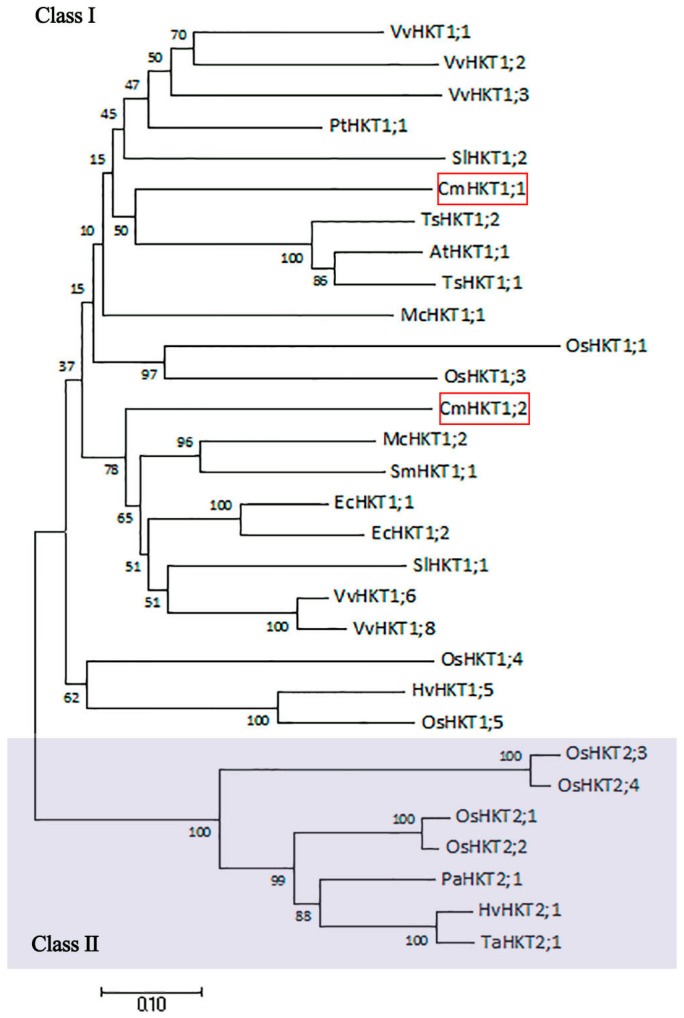
Phylogenetic relationship between pumpkin HKT proteins and other plant HKTs. The evolutionary history was inferred using the neighbor-joining method. Evolutionary analyses were conducted in MEGA7 [30]. The scale bar corresponds to a distance of 0.10 substitutions per site. Bootstrap values are indicated adjacent to the corresponding node. The protein accession numbers as listed in the GeneBank database are: AtHKT1;1 NP_567354; EcHKT1;1 AAF97728; EcHKT1;2 AAD53890; HvHKT1;5 ABK58096; HvHKT2;1 CAJ01326; McHKT1;1 AAK52962; McHKT1;2 AAO73474; OsHKT1;1 CAD37183; OsHKT1;3 CAD37185; OsHKT1;4 CAD37197; OsHKT1;5 BAB93392; OsHKT2;1 BAB61789; OsHKT2;2 BAB61791; OsHKT2;3 CAD37187; OsHKT2;4 CAD37199; PaHKT2;1 BAE44385; PtHKT1;1 XP_002325229.1; SlHKT1;1 NP_001295273.1; SlHKT1;2 NP_001289833.1; SmHKT1;1 AAS20529.2; TaHKT2;1 AAA52749; TsHKT1;1 JQ063120; TsHKT1;2 BAJ34563; VvHKT1;1 CAO64083; VvHKT1;2 CAO64075; VvHKT1;3, CAO64081; VvHKT1;6 CAO64069; and VvHKT1;8 CAO64071. At, *Arabidopsis thaliana*; Cm, *Cucurbita moschata*; Ec, *Eucalyptus camaldulensis*; Hv, *Hordeum vulgare*; Mc, *Mesembryanthemum crystallinum*; Os, *Oryza sativa*; Pa, *Phragmites australis*; Pp, *Physcomitrella patens*; Pt, *Populus trichocarpa*; Sl, *Solanum lycopersicum*; Sm, *Suaeda maritima*; Ta, *Triticum aestivum*; Ts, *Thellungiella salsuginea*; Vv, *Vitis vinifera*.

To understand the phylogenetic relationship between CmHKT1;1 and HKT-type proteins of other reported plants, we constructed a phylogenetic tree using the amino acid sequence of 28 known HKT proteins from various organisms. The phylogenetic tree shows that the gene families were split into two major families, namely, class I and class II. The pumpkin HKT genes belong to class I HKTs, typical of dicotyledonous plants (Figure 2). This finding suggests that CmHKT1;1 is a Na^+^-selective transporter.

### 2.2. NaCl Stress-Induced CmHKT1;1 Upregulated in Pumpkin

**Figure 3:**
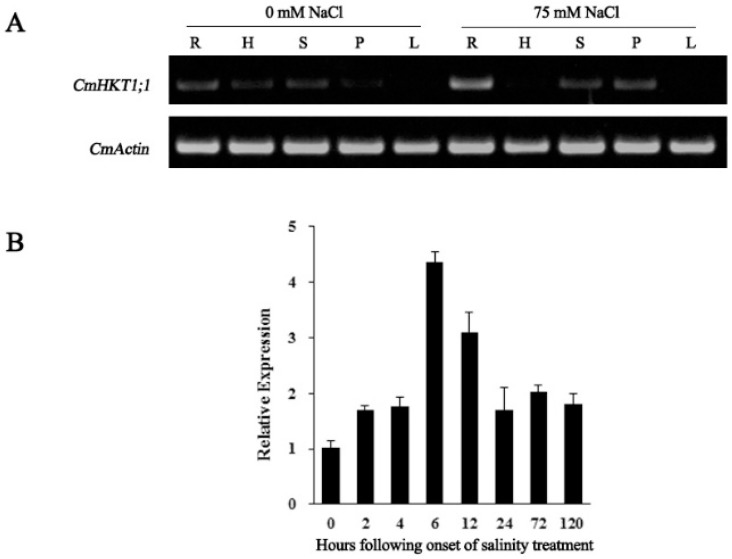
Expression pattern of *CmHKT1;1* in pumpkin under salinity stress. (**A**) RT-PCR analysis of *CmHKT1;1* expression in pumpkin. RNA was extracted from the root (R), hypocotyl (H), stem (S), petiole (P), and leaf (L) of the plants after 24 h of 75 mM salt treatment, and the reverse transcription products were amplified. *CmHKT1;1* expression in plants without NaCl was used as control. (**B**) Time-course expression analysis of *CmHKT1;1* in response to NaCl (75 mM) treatment by qRT-PCR in pumpkin roots. The *Actin* gene was used as the internal control. Error bars represent SE (*n* = 3).

We subsequently investigated the expression patterns of *CmHKT1;1*. The transcript level of *CmHKT1;1* in different tissues of pumpkin under NaCl stress was detected by RT-PCR. Pumpkin plants were treated with 75 mM NaCl for 24 h and then divided into five parts: root, hypocotyl, stem, petiole, and leaf. Under control condition, *CmHKT1;1* was expressed at a relatively low level in all tissues examined, but salinity significantly increased its expression in the stem and petiole, especially in the roots (Figure 3A).

Plant roots are the primary site of perception and injury for salt stress. In cucumber plants grafted with pumpkin as the rootstock, the roots contribute significantly to salt tolerance [24]. In the present study, qRT-PCR was performed to measure *CmHKT1;1* expression level in pumpkin roots under saline conditions. Results showed that the transcript level of *CmHKT1;1* in the roots was approximately 1.5- to 4.5-fold greater than that in the control, peaking at 6 h following onset (Figure 3B). This result indicates that *CmHKT1;1* expression is induced by salt stress in pumpkin roots, implicating the key role of this gene in the salt tolerance of pumpkin.

### 2.3. CmHKT1;1 Is Expressed at the Plasma Membrane of Stelar Cells in Pumpkin Roots

**Figure 4:**
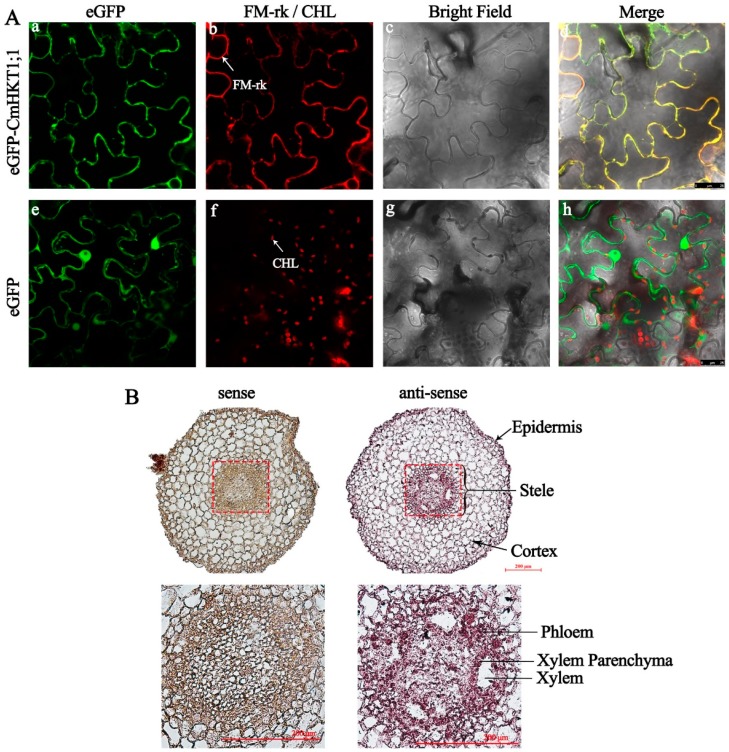
Membrane and cell-type localization of CmHKT1;1. (**A**) Subcellular localization of eGFP-CmHKT1;1 in the pavement cells of *N. benthamiana* leaves. **a**, Green fluorescence from eGFP-CmHKT1;1. **b**, Red fluorescence from PM-rk-labeled membrane in eGFP-CmHKT1;1 expressing cells. **c**, Bright field image of the cell shown in a and b. **d**, Overlay image of a, b, and c. **e**, Green fluorescence from the free eGFP. **f**, Chlorophyll autofluorescence (CHL)-derived red fluorescence in eGFP-expressing cells. **g**, Bright field image of the cell shown in e and f. **h**, Overlay image of e, f, and g. (**B**) Localization of *CmHKT1;1* mRNA in pumpkin root by *in situ* hybridization. Three true leaves aged pumpkin seedlings were subjected to 75 mM NaCl for 24 h before tissue collection. Cells in which transcript is present stain red-brown. Magnified views are shown below. Left panel, Sense probe. Right panel, antisense probe. The indication marks on the root cross section show the different tissues. Bars = 200 µm.

A transient protein expression analysis was performed to determine the localization of the CmHKT1;1 protein in plant cells. We fused eGFP at the N-terminus end of CmHKT1;1 and placed it under the control of the CaMV35S promoter, and then injected the fusion protein of eGFP-CmHKT1;1 into *Nicotiana benthamiana* epidermal cells. Confocal microscopy analyses showed the presence of eGFP fluorescence at the cell periphery (Figure 4Aa). The green fluorescence clearly overlapped with the red fluorescence from the plasma membrane marker (PM-rk CD3-1007) (Figure 4Ab), indicating that CmHKT1;1 localized at the plasma membrane.

The tissue specific expression of *CmHTK1;1* in the roots was investigated by *in situ* hybridization technique in pumpkin plants under salt stress. Cross sections of the roots were hybridized with antisense or sense RNA probes specific for *CmHKT1;1* transcript. In the roots, labeling of *CmHKT1;1* expression with the antisense probe was the strongest in the stele, particularly within the xylem parenchyma. The control with the *CmHKT1;1* sense probe did not lead to any significant labeling (Figure 4B).

### 2.4. CmHKT1;1 Prefers Na^+^ Transport over K^+^ Transport

**Figure 5:**
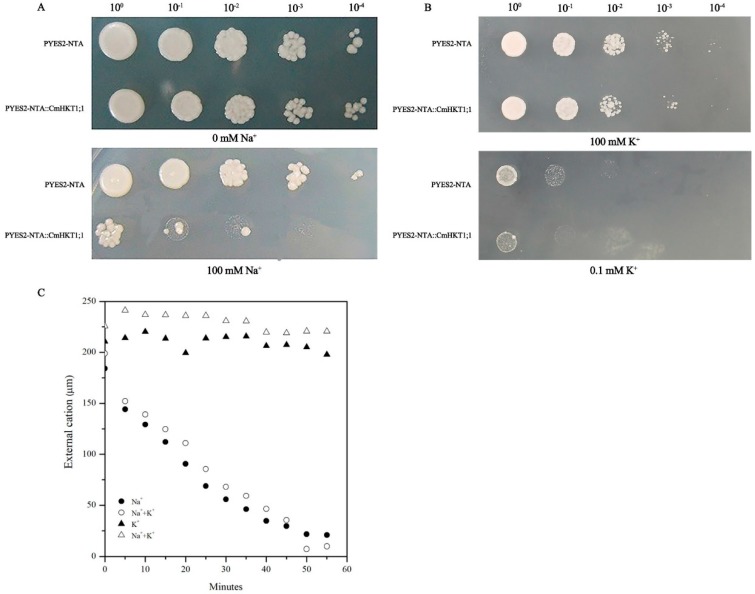
Functional characterization of CmHKT1;1 expressed in *S. cerevisiae*. (**A**) Growth inhibition tests of *S. cerevisiae* G19 (*Mat a, his3, leu2, ura3, trp1, ade2, and ena1::HIS3::ena4*) expressed the *CmHKT1;1* gene and empty vector. (**B**) K^+^ uptake complementation test in WΔ6. Growth of yeast strain WΔ6 (*Mat a ade2 ura3 trp1 trk1D::LEU2 trk2D::HIS3*) cells harboring the *CmHKT1;1* and empty vector. All transformants were grown on AP medium containing the indicated concentration of KCl and NaCl. (**C**) Time course of Na^+^ and K^+^ depletion in WΔ6 yeast cells expressing *CmHKT1;1*. K^+^-starved cells were suspended in testing buffer (10 mM MES-Ca^2+^, pH 6.0) supplemented by 2% galactose, and samples were taken at intervals after the addition of NaCl (200 mM) or KCl (200 mM). Depletion of K^+^ and Na^+^ was determined by atomic absorption spectrophotometry. Changes in Na^+^ concentration (circles) in the absence (closed circles) or presence of K^+^ (open circles), and changes in K^+^ concentration in the absence (closed triangles) or presence of Na^+^ (open triangles) were recorded.

*CmHKT1;1* was expressed in yeast heterologous expression systems to assess the Na^+^ and K^+^ transport activities of CmHKT1;1. Sodium uptake was examined by a growth inhibition test using the budding yeast (*Saccharomyces cerevisiae*) mutant strain G19, a salt hypersensitive yeast mutant. G19 transformed with CmHKT1;1 or an empty vector control plasmid was grown on agar plates with or without additional 100 mM NaCl in AP medium. The growth of yeast transformed with CmHKT1;1 was similar to that of the empty vector control when grown in AP media without NaCl. Treatment with 100 mM NaCl significantly suppressed the growth of CmHKT1;1-expressing yeast compared with the empty vector controls (Figure 5A). The reduced growth phenotype observed for the yeast expressing *CmHKT1;1* was similar to that observed for the yeast expressing the *A. thaliana* Na^+^ transporter gene *AtHKT1;1* in a previous study [3,6]. These results show that *CmHKT1;1* expression causes a Na^+^ hypersensitivity phenotype in yeast, indicating that CmHKT1;1 is likely to function as a Na^+^-permeable transporter similar to AtHKT1;1.

K^+^ uptake property was examined by a yeast functional complementation test. *CmHKT1;1* and the vector were expressed in the K^+^ uptake-deficient yeast (*S. cerevisiae*) mutant strain WΔ6. WΔ6 was transformed with *CmHKT1;1*, and an empty vector control plasmid was grown on agar plates with 100 mM and 0.1 mM KCl in AP medium. KCl at 100 mM was used as the control. Under 100 mM KCl, the WΔ6 cells expressing *CmHKT1;1* showed normal growth conditions similar to WΔ6 cells containing the vector alone (Figure 5B). Under the low-potassium condition (0.1 mM K^+^), the growth of the yeast was inhibited, and *CmHKT1;1* expression cannot improve this growth (Figure 5B). After 4 h K^+^ starvation, WΔ6 cells expressing *CmHKT1;1* were able to deplete external Na^+^, and this Na^+^ depletion was unaffected by the presence of K^+^ (Figure 5C). These results indicate that CmHKT1;1 is a Na^+^-selective transporter, i.e., without transport activity for K^+^.

### 2.5. Ectopic Expression of CmHKT1;1 in Cucumber Confers Salt Tolerance in Transgenic Plants

**Figure 6:**
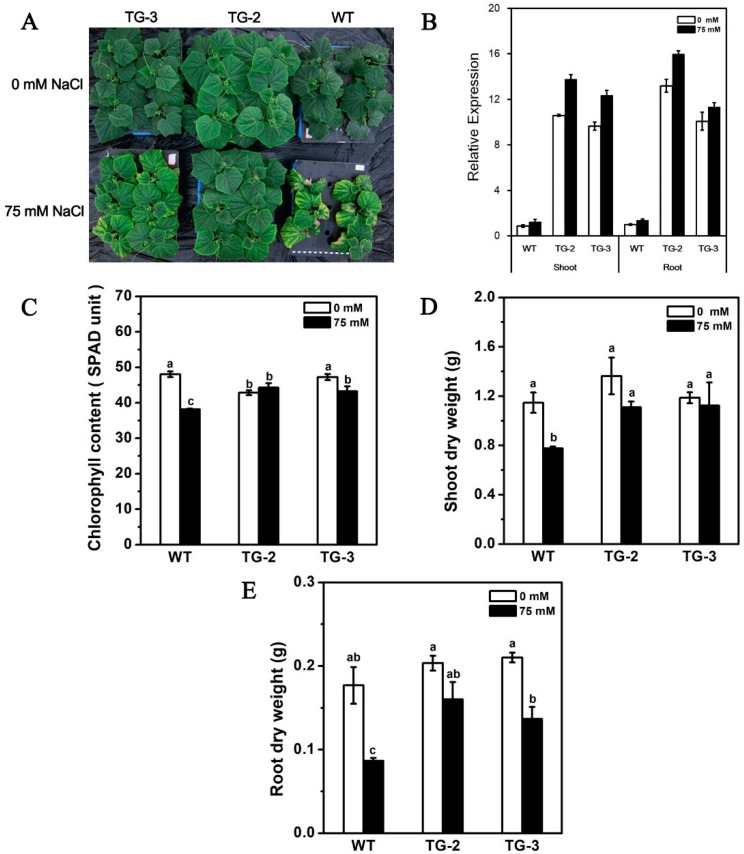
Effect of NaCl treatment on growth in transgene *CmHKT1;1* cucumber. (**A**) Three true leaves aged transgene *CmHKT1;1* cucumber cultivated in hydroponics with 75 mM NaCl for 7days. (**B**) qRT-PCR was used to analyze the expression of *CmHKT1;1* in WT and *CmHKT1;1* transgenic plants. (**C**) Chlorophyll content. (**D**) Shoot dry weight. (**E**) Root dry weight. WT, wild type. TG, transgene *CmHKT1;1*. Each value is the mean of three replications ± standard error of the means. Columns with different letters indicate they are significantly different at the *p* < 0.05 level (Duncan test).

Transgenic cucumber was generated to evaluate the influence of *CmHKT1;1* on cucumber salt tolerance. In total, we produced seven independent transgenic (TG) positive plants, but only four independent transgenic (TG) lines ectopically expressed *CmHKT1;1* (Supplemental Figure S2). In the T2 progeny, two independent transgenic lines (TG-2 and TG-3) were selected for further study (Figure 6B). 

Transgenic and WT plants after sowing for 25 days were cultivated hydroponically in aerated nutrient solution with 75 mM NaCl for 7 days or 0 mM NaCl as the control. Results showed a significant phenotypic difference between the WT and transgenic plants (Figure 6A). The transgenic plants showed better salt tolerance, higher chlorophyll content, and maximum dry weight of the shoots and roots than the non-transgenic plants (Figure 6C–E), which agree with our previous results [31]. These results indicate that the transgenic plants are tolerant to salt stress and that CmHKT1;1 plays a positive role in salt tolerance.

**Figure 7:**
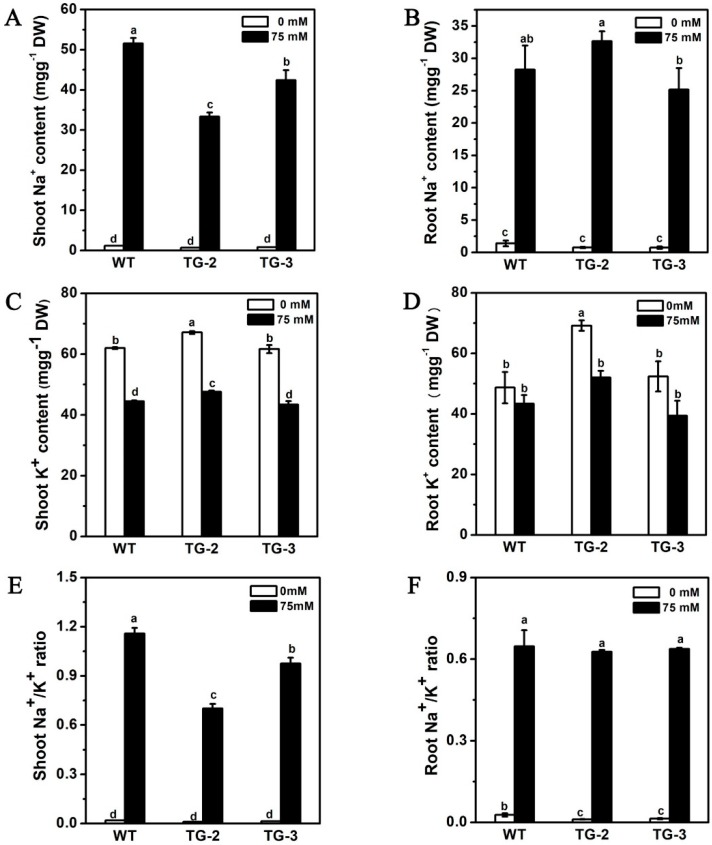
Shoot and root Na^+^/K^+^ content in control and NaCl-treated transgene *CmHKT1;1* cucumber. Transgene *CmHKT1;1* cucumber cultivated in hydroponics with 75 mM NaCl for 7 days. Shoot Na^+^ content (**A**), K^+^ content (**C**)**,** and Na^+^/K^+^ ratio (**E**). Root Na^+^ content (**B**), K^+^ content (**D**), and Na^+^/K^+^ ratio (**F**). WT, wild type. TG, transgene *CmHKT1;1*. Each value is the mean of three replications ± standard error of the means. Columns with different letters indicate they are significantly different at the *p* < 0.05 level (Duncan test).

We also measured the Na^+^ and K^+^ concentrations in the shoots and roots to investigate the effect of *CmHKT1;1* on Na^+^ accumulation in plants. Without salt stress, the Na^+^ concentration in the WT and transgenic lines of cucumber was very low, with no obvious differences in the roots and shoots. However, the K^+^ content in the roots and shoots of the transgenic lines was slightly higher than that in roots and shoots of the WT (Figure 7A–D). After 7 days of 75 mM NaCl treatment, the transgenic plants showed significantly reduced Na^+^ content, slightly increased K^+^ content, and notably decreased Na^+^/K^+^ ratio in the shoots compared with the WT plants (Figure 7A,C,E). Meanwhile, the Na^+^ content, K^+^ content, and Na^+^/K^+^ ratio in the roots of the transgenic plants and WT showed no significant difference under saline conditions, although TG-2 had a higher Na^+^ content than WT (Figure 7B,D,F). These results suggest that CmHKT1;1 mainly affects Na^+^ accumulation and regulates Na^+^/K^+^ ratio in the shoots.

### 2.6. CmHKT1;1 Transgenic Cucumber as Rootstock Improve the Growth of Wild-Type Cucumber Scion under Salt Stress

**Figure 8:**
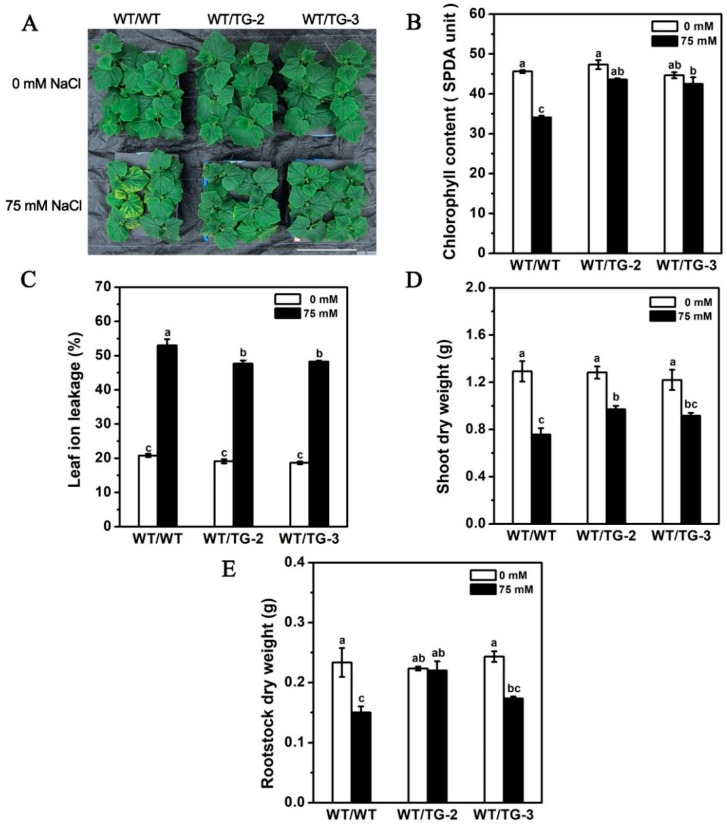
Effect of NaCl treatment on growth in grafted seedlings with transgene *CmHKT1;1* cucumber as rootstock. (**A**) Grafted cucumber seedlings were cultivated in hydroponics with 75 mM NaCl for 7 days. (**B**) Chlorophyll content. (**C**) Leaf ion leakage. (**D**) Shoot dry weight. (**E**) Root dry weight. WT, wild type. TG, transgene *CmHKT1;1*. WT/WT, WT as the scion and rootstock; WT/TG, WT as the scion, TG as the rootstock. Each value is the mean of three replications ± standard error of the means. Columns with different letters indicate they are significantly different at the *p* < 0.05 level (Duncan test).

A previous study on *Arabidopsis* found through reciprocal grafting experiments that loss of *AtHKT1* expression in the roots is responsible for elevated shoot Na^+^ [32]. Thus, in the present study, we investigated whether *CmHKT1;1* overexpression in the roots decreases shoot Na^+^ accumulation. We used two *CmHKT1;1* transgenic lines and WT cucumber seedlings to construct two grafting combinations, namely, self-grafting (WT as scion and WT as rootstock, WT/WT) and heterologous grafting (WT as scion and *CmHKT1;1* transgenic cucumber as rootstock, WT/TG). Then, these lines were subjected to salt treatment. Under standard conditions, the growth of all the plants was almost the same. After 75 mM NaCl treatment for 7 days, the WT/WT plants displayed obvious salt damage symptoms (Figure 8A), significantly lower chlorophyll content, and significantly higher leaf ion leakage compared with the WT/TG combinations (Figure 8B,C). The dry weight of shoot and rootstock in WT/TG-2 was also significantly higher than that in WT/WT, whereas that in WT/TG-3 was higher than that in WT/WT, but the difference was not significant (Figure 8D,E). These findings indicate that the transgenic cucumber as rootstock can relieve the salt damage of the WT shoots.

**Figure 9:**
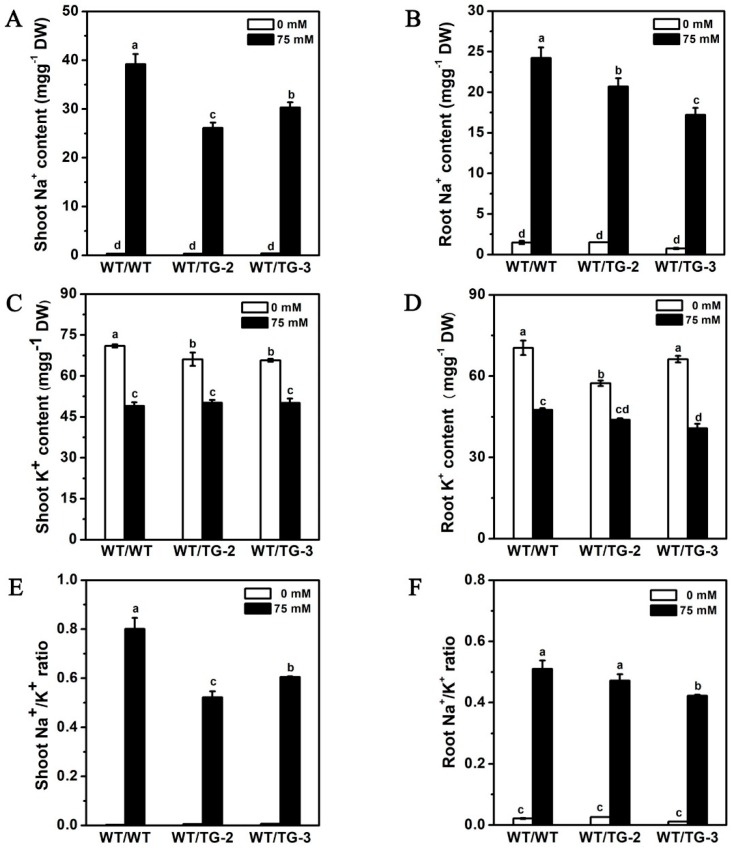
Shoot and root Na^+^/K^+^ content in control and NaCl-treated grafted seedlings with transgene *CmHKT1;1* cucumber as rootstock. Grafted cucumber seedlings cultivated in hydroponics with 75 mM NaCl for 7 days. Shoot Na^+^ content (**A**), K^+^ content (**C**), and Na^+^/K^+^ ratio (**E**). Root Na^+^ content (**B**), K^+^ content (**D**), and Na^+^/K^+^ ratio (**F**). WT, wild type. TG, transgene *CmHKT1;1* cucumber. WT/WT, WT as the scion and rootstock; WT/TG, WT as the scion, TG as the rootstock. Each value is the mean of three replications ± standard error of the means. Columns with different letters indicate they are significantly different at the *p* < 0.05 level (Duncan test).

The Na^+^ and K^+^ contents were also measured, and the shoot Na^+^ content in WT/TG was significantly lower than that in WT/WT. The K^+^ contents were almost similar in all the combinations. The Na^+^/K^+^ ratio in WT/TG was obviously lower than that in WT/WT (Figure 9A,C,E). Interestingly, the Na^+^ content in the roots of WT/TG was significantly lower than that in the roots of WT/WT. It was very different with the Na^+^ content in the roots of self-grafted *CmHKT1;1* transgenic seedlings. The K^+^ content and the root Na^+^/K^+^ ratio were lower in WT/TG than in WT/WT, although the difference was not significant (Figure 9B,D,F).

## 3. Discussion

### 3.1. CmHKT1;1 Is a Pumpkin Na^+^-Selective Class I HKT

HKTs are crucial for salt tolerance in plants along with other transporters. They are responsible for ion homeostasis and Na^+^ distribution within plants [33,34]. HKT transporters are divided into two groups, class I and class II, depending on their roles in K^+^ and Na^+^ transport within plants [7,34]. The presence of Ser instead of Gly in the PA-loop is a key feature that determines preferential Na^+^ conductance in class I HKTs [5,33,34,35,36]. We isolated two HKT1-like isoforms from pumpkin, namely, CmHKT1;1 and CmHKT1;2. Following sequence and phylogenetic analysis, both are classified as class I HKTs (Figure 2). These class HKTs have been described as low-affinity and specific Na^+^ transporters responsible for unloading Na^+^ from the xylem, thus preventing Na^+^ accumulation in the shoots [3,6,8,10,12,37]. However, TsHKT1;2 in the halophytic *Thelungiella salsuginea* belongs to class I, showing strong K^+^ transporter activity and selectivity over Na^+^ [29]. K^+^ transport capacity was attributed to the presence of two aspartic residues, D207 and D238, in the transmembrane (M2B) and pore (PB) domains. In these locations, Asn (N) residues were found in *Arabidopsis* and all other known plant sequences [29]. These residues are absent in the sequence of any pumpkin isoforms, indicating that both are similar in sequence to typical Na^+^ transporters (Figure 2). Following heterologous expression experiments in Na^+^-sensitive yeast mutant cells (G19) and endogenous K^+^ transport-defective yeast mutant cells (WΔ6), we can conclude that pumpkin CmHKT1;1, similar to AtHKT1;1 [3], is a Na^+^ transporter and not a K^+^ transporter (Figure 5). With respect to CmHKT1;2, long fragment amino acids lost in the N-terminal were observed, and the first M1A-PA-M1B transmembrane domains were incomplete (Figure 1). Even the mRNAs were not differentially expressed under salt stress and thus were not given focus in this study.

HKT family proteins primarily promote the exclusion of Na^+^ from the leaves via the withdrawal of Na^+^ from root-to-shoot-flowing xylem sap [6,10,12,38]. In monocot plants, HKT1 location was intensively studied, and analysis of transgenic rice lines containing the GUS reporter gene under the control of the *OsHKT1;1* promoter or *in situ* hybridization experiments showed that *OsHKT1;1* is expressed mainly in the phloem of leaf blades [39,40]. In bread wheat, tissue-specific expression of *TaHKT1;5-D* mRNA in the roots is predominantly observed by *in situ* PCR within the stele, particularly within xylem parenchyma and pericycle cells adjacent to the xylem vessels [13,38]. In *Sorghum bicolor*, *SbHKT1;4* is expressed in the roots, sheath, and leaf veins [41]. In the dicotyledonous plant *Arabidopsis*, tissue-specific expression analyses of *AtHKT1;1* showed its expression in the root stele and leaf vasculature [5]. Further biochemical immunolocalization analyses found that the AtHKT1;1 protein is targeted to the plasma membrane of xylem parenchyma cells suggesting that AtHKT1;1 functions in the removal of Na^+^ from the xylem sap [10]. In tomato, *in situ* PCR results showed that *SlHKT1;1* and *SlHKT1;2* are expressed in vascular bundle cells of leaves, but only *SlHKT1;2* expression could be detected in stellar cells of roots [42]. In the present study, *CmHKT1;1* was strongly expressed in the root system and significantly highly upregulated under Na^+^ stress (Figure 3). *In situ* hybridization showed that *CmHKT1;1* was mainly expressed in pumpkin root stellar cells (Figure 4). These results suggest that CmHKT1;1 is ideally localized to withdraw Na^+^ from the root xylem sap, thus reducing the root-to-shoot flow of Na^+^ and decreasing shoot Na^+^ accumulation.

### 3.2. CmHKT1;1 Enhances Salinity Tolerance in Transgenic Cucumber

Salt tolerance and Na^+^ exclusion mechanisms vary in different plant species. Previous studies used genetic engineering to address HKT function. In bread wheat, the RNAi lines reducing *TaHKT1;5-D* transcripts in planta increase leaf Na^+^ concentration [13]. In barley, loss of *HvHKT1;2* function in a mutant plant reduces Na^+^ influx and growth under saline conditions, and *HvHKT2;1* overexpression increases leaf Na^+^ and salt tolerance [43,44]. *AtHKT1;1* overexpression in the mature root stele of *A. thaliana* decreases Na^+^ accumulation in the shoots [9], and *AtHKT1;1*expression in the root cortex of rice increases salinity tolerance and decreases shoot Na^+^ accumulation [15]. In tomato, *SlHKT1;2* silencing promotes Na^+^ accumulation in the shoots and reduces plant growth under salt stress [42]. In woody plants, *VisHKT1;1* is a major gene controlling Na^+^ exclusion in grapevine rootstocks [45]. Interestingly, the constitutive expression of *AtHKT1;1* in WT *Arabidopsis* driven by the cauliflower mosaic virus 35S promoter causes high shoot Na^+^ accumulation and poor growth [9], and overexpressing *AtHKT1;1* in the mutant *athkt1-3* under the control of 35S, also suppresses plant growth [29]. However, overexpression of *AtHKT1* and *SlHKT1;2* in the *Arabidopsis* mutant *athkt1;1*, which is driven by the *AtHKT1* promoter region, a 5 kb DNA fragment upstream of the ATG start codon of the *AtHKT1;1* gene containing the promoter region, the tandem repeat, and the small RNA target region [46], could reduce Na^+^ accumulation in the shoots [47]. These results indicate that *HKT1* genes need to be expressed in a specific cell type or tissue in transgenic plants to improve salinity tolerance. However, transgenic barley plants overexpressing *HvHKT2;1,* which is driven by a strong constitutive ubiquitin promoter, demonstrate increased salt tolerance [44]. The novel soybean gene *GmHKT1;4* driven by the CaMV35S promoter enhances the root growth of the tobacco transgenic line and increases the salinity tolerance of tobacco [16]. Ectopic expression of the *Mesembryanthemum crystallinum* sodium transporter *McHKT2*, which encodes for the *Arabidopsis* sodium transporter ortholog *AtHKT1* driven by 35S, confers strong salt tolerance in *Arabidopsis* [48]. *SsHKT1;1* is an HKT1 homolog isolated from C3 halophyte *Suaeda salsa* L. and is a K^+^-selective transporter. Driven by 35S, SsHKT1;1 overexpression in *Arabidopsis* enhances salt tolerance and increases shoot K^+^ concentration [49]. In maize, two alternative splicing variants *ZmHKT1;1* confer salt tolerance in transgenic tobacco plants and decrease the Na^+^ content in the shoots [4]. In the present study, the ectopic expression of *CmHKT1;1* in cucumber enhanced the salt tolerance in transgenic cucumber and decreased Na^+^ accumulation in the shoots (Figure 6 and Figure 7). The functional differences in different species might be explained by the variations in amino acid sequences, and HKT proteins might enhance salt tolerance through different mechanisms. The regulatory network and the role in long-distance transport of HKTs remain unclear [50].

### 3.3. CmHKT1;1 Is Critical for Regulation of Shoot Na^+^ Accumulation in Grafted Cucumber through Limiting Na^+^ Transport from the Roots to the Shoots

Grafting onto tolerant rootstocks is a cost-effective and efficient approach to reduce the effect of salinity on some horticultural crops, such as tomato, cucumber, watermelon, melon, citrus, and grapevine [19,45,51,52,53,54]. The use of salt-resistant rootstocks can improve salinity tolerance in shoots. In *Arabidopsis*, mutant *hkt1* and WT *Col-0* reciprocal grafting experiments showed that the loss of *AtHKT1;1* expression in the roots increases the shoot Na^+^ content [32]. Reciprocal grafting between cucumber and pumpkin demonstrates the roles of the rootstock in determining Na^+^ accumulation in cucumber [24]. In the current study, we used the *CmHKT1;1* transgenic line as the rootstock and WT cucumber as the scion. Grafting of the plants decreased the Na^+^ content in the shoots and improved the growth of the grafted cucumber seedlings under salt stress (Figure 8 and Figure 9). 

The Na^+^ ion accumulation in the leaves is controlled predominantly by the genotype of the rootstock, and the characteristics of the rootstock to induce salt tolerance in the shoots depend on the salt tolerance mechanism of the shoot genotype [55]. In cucumber and tomato, which are the reciprocal grafted plants, the Na^+^ in the shoots and roots is also impacted by the rootstock and scion with different levels of salt tolerance [24,56]. Hence, the interaction between the scion and the rootstock is complicated. Previous studies on HKT function mainly focused on the changes in shoot Na^+^ content, and few studies demonstrated the changes in root Na^+^ content. In the present study, the *CmHKT1;1*-expressing cucumber had significant reduction in shoot Na^+^ content, relatively higher root Na^+^ content in TG-2, and lower Na^+^ content in TG-3 compared with the WT (Figure 7B). However, in the grafting combinations, the rootstock Na^+^ content of the transgenic line was significantly lower than that of the WT rootstock under salt stress (Figure 9B). Whether the different results due to the different genotypes of the shoot needs further investigation in the future. 

In conclusion, CmHKT1;1 plays an important role in the Na^+^ homeostasis and salinity tolerance of pumpkin, especially in the grafted cucumber plants. The finding may be useful for engineering salt tolerance in cucurbit crops.

## 4. Materials and Methods 

### 4.1. Plant Material, Growth Conditions, and Stress Treatments

Cucumber (*C. sativus* L., Xintaimici) and pumpkin (*Cucurbita maxima × C. moschata*, Chaojiquanwang) were used in the current study. For the grafting experiment, rootstock and scion seeds were soaked in 55 °C water for 12 h, incubated at 30 °C until germination, and then sown in a mixture of peat/vermiculite (3/1, *v*/*v*). Seeds as the scion were soaked and sown 7 days later. Grafting was performed by hole-insertion grafting [57]. After 20 days, uniform seedlings were cultivated in plastic boxes (320 mm × 240 mm × 140 mm) hydroponically in a chamber with a controlled environment. Each container with 9 L of full-strength Hoagland solution contained six plants [58]. After 3 days, salt stress (75 mM NaCl) was applied. During the hydroponic cultivation, a 16/8 h light/dark cycle and an air temperature of 28 °C (day) and 18 °C (night) were adopted in the controlled environment chamber. The relative humidity was set at 70% (day) and 85% (night), and the photon flux density was 350 μmol m^−2^ s^−1^. The nutrient solutions were renewed at an interval of 3 days and were continuously aerated by an air pump. The roots and the shoots were harvested separately according to experimental requirement.

### 4.2. Transient Gene Expression in Tobacco

To analyze the subcellular localization of CmHKT1;1, we amplified the coding sequence of the pumpkin CmHKT1;1 by PCR with gene-specific primers (Supplementary Table S1) and inserted it into the StuI site of the pH7LIC5.0-N-eGFP vector by using ClonExpress II One Step Cloning Kits (Vazyme, Nanjing, China ) to generate 35S::eGFP-CmHKT1;1 fusion protein. The resulting vectors were then introduced into *Agrobacterium* strain GV3101 and delivered into tobacco (*N. benthamiana*) leaves via *Agrobacterium*-mediated transformation [59]. Meanwhile, the mCherry-labeled plasma membrane marker plasmid PM-rk CD3-1007 [60] was used for co-localization. The pH7LIC5.0-N-eGFP empty vector expressing untargeted GFP was used as a control. Two days after infiltration, cells expressing fluorescent protein fusions were observed via laser scanning confocal microscopy (Leica TCS-SP8, Solms, Germany).

### 4.3. Cation Uptake Experiments in Yeast Cells

The coding sequence of the pumpkin CmHKT1;1 was amplified by PCR with gene-specific primers (Supplementary Table S1) and inserted into the HindIII site of the pYes2-NTA vector under the *GAL1* promoter. The yeast salt hypersensitive mutant G19 strain (*Mata, his3, leu2, ura3, trp1, ade2, and ena1::HIS3::ena4*) [61,62] was used for yeast growth inhibition assay. The yeast K^+^ uptake-deficient mutant strain WΔ6 (*Mata ade2 ura3 trp1 trk1D::LEU2 trk2D::HIS3*) [63] was used for functional complementation and Na^+^/K^+^ transport assays. The plasmids containing CmHKT1;1 cDNA and an empty vector were transformed into G19 and WΔ6 in accordance with the manufacturer’s instructions (Clontech, Dalian, China). For both complementation and Na^+^ sensitivity analyses, the transformations were cultured in arginine phosphate (AP) medium [64] with the indicated concentrations of KCl and NaCl supplemented in each plate, and gene expression was induced by adding 2% (*w*/*v*) galactose. Overnight-cultured yeast was adjusted to an optical density of 0.7. Then, 5 μL of fivefold serial dilutions of cell solution was spotted on AP medium at 30 °C for 4 days. 

For Na^+^ uptake assays in yeast cells, we used the *CmHKT1;1*-transformed WΔ6. The K^+^-starved cells were previously obtained by transferring actively growing cells in 50 mM K^+^ AP medium to K^+^-free AP medium and then incubated for 4 h. The K^+^-starved cells were suspended in the testing buffer (10 mM MES-Ca^2+^, pH 6.0) plus 2% galactose, and samples were taken at intervals after the addition of the tested cation. Na^+^ uptake was carried out using the depletion procedure [63,65,66]. Na^+^/K^+^ was determined by using an atomic absorption spectrophotometer (Varian spectra AA 220, Varian, Palo Alto, CA, USA). All experiments were repeated at least three times.

### 4.4. Cucumber Transformation

For obtaining overexpression plant lines, the ORF of *CmHKT1;1* was cloned and ligated into the XhoI and XbaI restriction sites of the binary expression vector pHellsgate 8 under the control of the CaMV 35S promoter. Then, the constructed vector was transferred into *A. tumefaciens* strain LBA4404. The cucumber inbred line “Xintaimici” was used for transformation in accordance with a previously described procedure [67,68]. In brief, sterilized cucumber seeds were sown on Murashige and Skoog (MS) medium [69] containing 30 g L^−1^ sucrose and 2.5 g L^−1^ gellan gum powder (G434, PhytoTechnology Laboratories™, Shawnee Mission, KS, USA). At 1 day after the seeds germinated, the growing points and the upper halves of the cotyledons far away from the growing points were removed, and the other halves of cotyledons were dipped in the *A. tumefaciens* solution suspension containing MS liquid medium (OD_600_ = 0.3–0.5) for 15–20 min. These explants were placed upside down on MS medium containing 0.5 mg L^−1^ 6-benzylaminopurine and 1 mg L^−1^ abscisic acid and co-cultured for 2 days at 28 °C in the dark. Subsequently, the tissues were transferred onto MS medium containing 0.5 mg L^−1^ 6-benzylaminopurine, 1 mg L^−1^ abscisic acid, 25 mg L^−1^ kanamycin, and 500 mg L^−1^ carbenicillin (Sigma-Aldrich, Shanghai, China) and then cultured for 14–20 days at 28 °C, 100 µM m^−2^ s^−1^ photosynthetic photon flux density. The shoots with kanamycin resistance were cut and cultured in MS medium with 50 mg L^−1^ kanamycin and 200 mg L^−1^ carbenicillin for development into whole plants. After the plants grew well in the medium, the transgenic plants were transferred to the pots with sterile substrate. Regenerated plants were screened by PCR (the primers are listed in Supplementary Table S1), and the positive plants were planted in the plastic greenhouse for the next generation. Seedlings of T2 generation were used for NaCl treatment and grafting.

### 4.5. RT-PCR and Quantitative Real-Time PCR

Total RNA was extracted using the TransZol reagent (Transgen, Beijing, China), and first-strand complementary DNA (cDNA) was synthesized using a HiScript 1st Strand cDNA Synthesis Kit (Vazyme, Nanjing, China) in accordance with the manufacturer’s protocol. For RT-PCR, the *Actin* gene and *18S* were used as internal standards to normalize the expression data for the target genes. For quantitative RT-PCR, a TransStart Green qPCR SuperMix (Transgen, Beijing, China) was used on an ABI QuantStudio 7 Flex real-time PCR system. Expression levels of genes were determined from three independent biological replicates. The pumpkin and cucumber *Actin* genes were used as internal positive controls. The relative expression levels of the target genes were analyzed using the 2^−ΔΔCt^ method [70]. The used primer pairs are described in Supplemental Table 1.

### 4.6. Determination of Plant Dry Weight, Na^+^, and K^+^ Concentrations

Five plants per treatment were harvested, washed thrice with deionized water to eliminate salt adhering, and then dried with tissue paper. The plants were divided into the shoots (the part above the cotyledon) and the roots (the part below the cotyledon). Both parts were placed in a forced air oven at 105 °C for 15 min and at 70 °C for 72 h to determine their dry weights.

For determination of Na^+^ and K^+^ concentrations, the dried roots and shoots were ground to powder, weighed, and then digested in H_2_SO_4_-H_2_O_2_ (5:1) for 0.5 h at 400 °C for elemental extraction. The concentrations of Na^+^ and K^+^ were determined in appropriately diluted samples by using an atomic absorption spectrophotometer (Varian spectra AA 220, Varian, Palo Alto, CA, USA).

### 4.7. Determination of Chlorophyll Content and Electrolyte Leakage

The leaf relative chlorophyll content was measured using a SPAD-502 Chlorophyll Meter (Minolta Camera Co., Ltd., Osaka, Japan).

The second fully expanded leaves were collected for electrolyte leakage measurement after NaCl treatment in accordance with a previously described method [71]. In brief, leaf samples were cut into 1 cm^2^ small rounds by a hole punch, one for 12 pieces, rinsed with deionized water, and then shaken for 3 h at room temperature. Conductivity was then measured as EL1 by an electrical conductivity meter (SG23; Mettler Toledo, Shanghai, China). Then, the leaf small rounds were boiled for 15 min, and conductivity was measured as total conductivity (EL2). Electrolyte leakage (%) = EL1/EL2 × 100.

### 4.8. In Situ Hybridization

Roots of pumpkin seedlings that have been treated with 75 mM NaCl for 24 h were cut into small sections and fixed in 3.7% FAA (5% acetic acid, 50% ethanol, and 3.7% formaldehyde) for 16 h at 4 °C. After dehydration through an ethanol series, tissues were embedded in Paraplast Plus (Fisher, Hampton, NH, USA) and sectioned at 10 μm thickness using a Leica Biosystems RM2265 Fully Automated Rotary Microtome (Leica Microsystems GmbH, Wetzlar, Germany). A gene-specific region of the coding region (77–785) of *CmHTK1;1* was amplified by PCR from the pumpkin cDNA and then transcribed in vitro under SP6 or T7 promoter with RNA polymerase using the DIG RNA labeling kit (Roche, Basel, Switzerland). The transcript was prepared for the DIG-labeled RNA antisense or sense probe. *In situ* hybridization was performed as previously described [72]. Images were obtained using a Nikon Eclipse 80i microscope. 

## Supplementary Materials

Supplementary materials can be found at http://www.mdpi.com/1422-0067/19/9/2648/s1.
