# Supplementary material for: Pumpkin CmHKT1;1 Controls Shoot Na+ Accumulation via Limiting Na+ Transport from Rootstock to Scion in Grafted Cucumber
Source: Int J Mol Sci. 2018 Sep 6;19(9):2648. doi: 10.3390/ijms19092648 (PMC6165489; doi:10.3390/ijms19092648)
Supplement: Supplementary file 1 [file ijms-19-02648-s001.pdf]

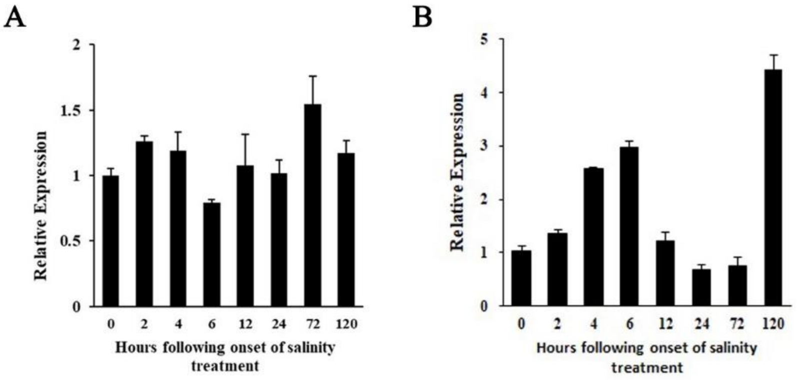

Supplementary Figure S1. Expression of CmHKT1;2 and CsHKT1;1 in response to NaCl. (A) Time-course expression analysis of *CmHKT1;2* response to NaCl (75 mM) treatment by qRT-PCR in pumpkin roots. The *Actin* gene was used as an internal control. Error bars represent SE (n = 3). **(B)** Time-course expression analysis of *CsHKT1;1* response to NaCl (75 mM) treatment by qRT-PCR in cucumber roots. The *Actin* gene was used as an internal control. Error bars represent SE (n = 3).

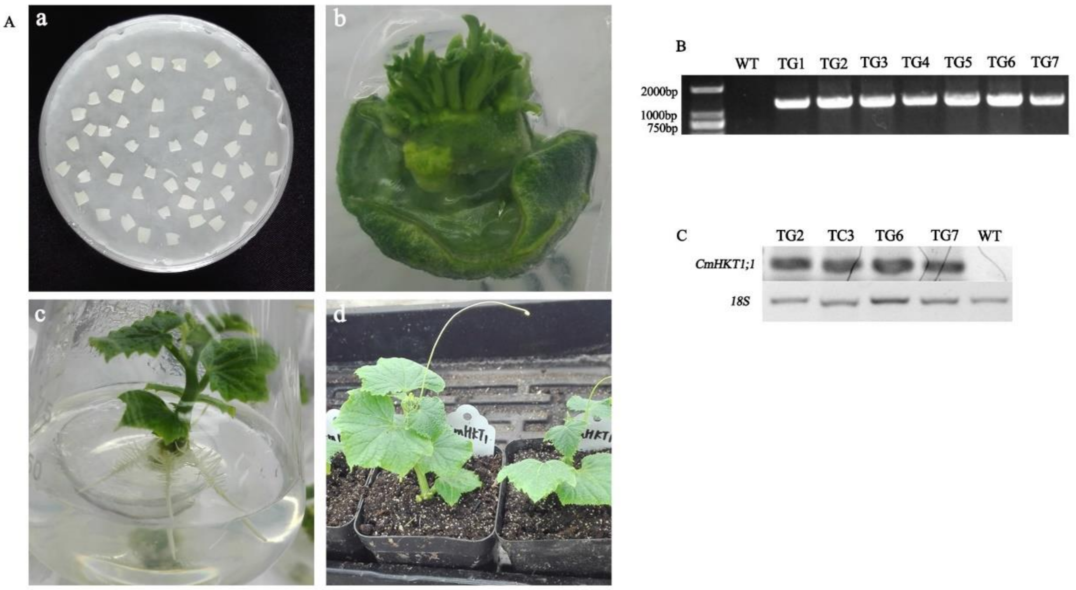

Supplementary Figure S2. Overexpression of *CmHKT1;1* in cucumber. (A) *Agrobacterium tumefaciens* mediated transformation in cucumber. **a**, Lower halves of cucumber cotyledons used for transformation; **b**, Explants on shoot induction medium (containing antibiotics and hormones); **c**, Shoots with kanamycin resistance were cut and cultured in MS rooting medium with antibiotics for development into whole plants; **d**, Transgenic plants were transferred to the pots with sterile substrate for naturalizing. **(B)** Identification of the positive transgenic plants by

PCR, with primers forward *CmHKT1;1* primer and reverse primer of OCS terminator (Oct).

(C) RT-PCR analysis of *CmHKT1;1* transcripts in transgenic cucumber.

Supplemental Table S1. Primers used in the paper.

**Primers**	**Description**	**Sequence (5’- 3’)**
CmHKT1;1-F	CDS clone	ATGAAGAACTTACCACTCTATTTCCT
CmHKT1;1-R	CDS clone	CTATGATAGTTTCCATGCTTTCCCAC
CmHKT1;1-F	Transgenic line PCR	TCTCCAACTTCCAACTCCT
Oct-R	Transgenic line PCR	CGTCTCGCATATCTCATTAAAGC
CmHKT1;1-F	RT-PCR	TCTCCAACTTCCAACTCCT
CmHKT1;1-R	RT-PCR	CCGACAATAGATGTGAATACC
CmActin-F	RT-PCR	CTCTGGTGATGGTGTTAGTC
CmActin-R	RT-PCR	TGTAAGTAGTCTCGTGGATTC
CsHKT1;1-F	qRT-PCR	TCTCATCATCACCACCCTCA
CsHKT1;1-R	qRT-PCR	CCGAATCGACATGGTTTTCT
Cs18S-F	RT-PCR	CGAGTCTGGTAATTGGAATGAGTA
Cs18S-R	RT-PCR	CTACGAGCTTTTTAACTGCAACAA
CmHKT1;1-F	qRT-PCR	TGTCTGTGGTCGAAATGGAA
CmHKT1;1-R	qRT-PCR	GTTCTGGTGGGAGAGTTGGA
CmHKT1;2-F	qRT-PCR	TTGTTTCCGTCGTGTTTGAG
CmHKT1;2-R	qRT-PCR	AAACACTCCCAACCAACAGC
CmActin-F	qRT-PCR	CACTACACCGTTGGAAAGGAAA
CmActin-R	qRT-PCR	CAAAAGGAGGGAGCCGAGA
CsHKT1;1-F	qRT-PCR	TCTCATCATCACCACCCTCA
CsHKT1;1-R	qRT-PCR	CCGAATCGACATGGTTTTCT
CsActin-F	qRT-PCR	CCACGAAACTACTTACAACTCCATC
CsActin-R	qRT-PCR	GGGCTGTGATTTCCTTGCTC
CmHKT1;1-SP6	*in situ* probe	GATTTAGGTGACACTATAGAATGCTTGGCTCACCTCTGCTATT
CmHKT1;1-T7	*in situ* probe	TGTAATACGACTCACTATAGGGATCACCGAGTTGTTCTTCA
